# Belladonna in Asthma

**Published:** 1871-02

**Authors:** 


BELLADONNA IN ASTHMA.—The action of belladonna in asthma is twofold. It acts upon the vessels of the spinal cord, and diminishes its sensibility. Secondly, it acts upon the large pulmonary vessels, causing them to contract, and so stimulating the pulmonary circulation. Belladonna seems to cause contraction of the muscular fibres of all the large arteries, it also produces more rapid action of the heart.—*Medical Archives.*

